# 

**Published:** 1901-01

**Authors:** 


***NOTICE.—This JOURNAL and THE INTERNATIONAL JOURNAL OF SURGERY one year for $1.00 cash, strictly to new subscribers. No out-of-town checks accepted unless 10c. is added for cost of cashing. Strictly to new subscribers.***

### EDITORIAL NOTES AND COMMENTS.

The Business office of THE JOURNAL-RECORD is No. 64 Marietta Street.

The Editorial office is 318-319-320 The Prudential.

Address all Business communications to Dr. M. B. Hutchins, Mgr.

Make remittances payable to THE ATLANTA JOURNAL-RECORD OF MEDICINE.

On matters pertaining to the Editorial and Original Communications address Dr. Bernard Wolff, Atlanta.

Reprints of original articles will be furnished at cost price. Requests for the same should always be made *on the manuscript*.

We will present, post-paid, on request, to each contributor of an original article, twenty (20) marked copies of THE JOURNAL-RECORD containing such article.

### NOTICE TO SUBSCRIBERS AND ADVERTISERS.
### IMPORTANT.

On December 19, 1900, while under arrest, the office boy confessed to having been opening letters, addressed both to the Journal-Record and to Dr. M. B. Hutchins, in search of money. He stated that he took money found in letters and destroyed all letters opened. He destroyed a great many checks and P. O. orders, because they were useless to him, as the result of which we find our accounts against many subscribers and advertisers still open.

All parties who have made remittances in the past three months for which they have not got a receipt will please look up check or money order record and have duplicate sent us. Persons who have sent unregistered money have done so at their own risk, and will have to present proof of such remittance in order to have account credited.

M. B. HUTCHINS, Manager.

***NOTICE.—This JOURNAL and THE INTERNATIONAL JOURNAL OF SURGERY one year for $1.00 cash, to new subscribers. No out-of-town checks accepted unless 10c. is added for cost of cashing. Strictly to new subscribers.***

***NOTICE.—This JOURNAL and THE INTERNATIONAL JOURNAL OF SURGERY one year for $1.00 cash, to new subscribers. No out-of-town checks accepted unless 10c. is added for cost of cashing. Strictly to new subscribers.***

